# Characterization and Antimicrobial Activity of Amphiphilic Peptide AP3 and Derivative Sequences

**DOI:** 10.3390/antibiotics8010020

**Published:** 2019-03-06

**Authors:** Christina L. Chrom, Lindsay M. Renn, Gregory A. Caputo

**Affiliations:** 1 Department of Chemistry and Biochemistry, Rowan University, Glassboro, NJ 08028, USA; chromc95@rowan.edu (C.L.C.); rennl28@rowan.edu (L.M.R.); 2 Department of Molecular and Cellular Biosciences, Rowan University, Glassboro, NJ 08028, USA

**Keywords:** antimicrobial peptides, pore-forming peptides, fluorescence spectroscopy, membrane permeabilization

## Abstract

The continued emergence of new antibiotic resistant bacterial strains has resulted in great interest in the development of new antimicrobial treatments. Antimicrobial peptides (AMPs) are one of many potential classes of molecules to help meet this emerging need. AMPs are naturally derived sequences, which act as part of the innate immune system of organisms ranging from insects through humans. We investigated the antimicrobial peptide AP3, which is originally isolated from the winter flounder *Pleuronectes americanus*. This peptide is of specific interest because it does not exhibit the canonical facially amphiphilic orientation of side chains when in a helical orientation. Different analogs of AP3 were synthesized in which length, charge identity, and Trp position were varied to investigate the sequence-structure and activity relationship. We performed biophysical and microbiological characterization using fluorescence spectroscopy, CD spectroscopy, vesicle leakage assays, bacterial membrane permeabilization assays, and minimal inhibitory concentration (MIC) assays. Fluorescence spectroscopy showed that the peptides bind to lipid bilayers to similar extents, while CD spectra show the peptides adopt helical conformations. All five peptides tested in this study exhibited binding to model lipid membranes, while the truncated peptides showed no measurable antimicrobial activity. The most active peptide proved to be the parent peptide AP3 with the highest degree of leakage and bacterial membrane permeabilization. Moreover, it was found that the ability to permeabilize model and bacterial membranes correlated most closely with the ability to predict antimicrobial activity.

## 1. Introduction

Antimicrobial peptides (AMPs) are biomolecules conserved through evolution and found in all complex living organisms [1,2]. There is significant diversity in peptides that exhibit antimicrobial activity. One of the most well-studied classes are those which are ribosomally synthesized as part of the natural host defense mechanism [3]. They are widely used as a defense mechanism among multicellular organisms and are often produced to play an important role in innate immune systems and the host defense mechanism [4,5,6,7]. As such, bacteria were exposed to AMPs throughout millions of years of evolution, yet there has not been any significant development of resistance [8]. This low propensity for resistance and ability to kill or inhibit bacterial growth has made AMPs an ideal target for new drug discovery to help combat antibiotic resistant bacteria [1,8].

Naturally occurring antimicrobial peptides are gene-encoded, ribosomally synthesized polypeptides [9]. These peptides are usually short (less than 100 amino acid residues), have a positive net charge, amphipathic nature, and often exhibit a broad spectrum of activity against bacteria, viruses, and fungi [1,10,11]. Because of the wide sequence and structural diversity, AMPs are traditionally classified based on secondary structure conformations: α-helices, β-sheets, or random coil [2]. There are several well-known examples of antimicrobial peptides belonging to the families of the cathelicidins, defensins, thionins, cecropins, and magainins [12,13,14,15]. 

The mechanism of action for cationic antimicrobial peptides is not fully understood. However, many studies point to the mechanism of action beginning with the positively charged peptide interacting with negatively charged bacterial cell surface [2,8,11]. This interaction is followed by the insertion of the peptide into the membrane leading to disruption. This process is thought to be driven by the partitioning of hydrophobic amino acid side chains in the nonpolar core of the bacterial membrane [16,17,18]. Although it is difficult to investigate in bacterial systems, using model membrane vesicle approaches have shown the binding and insertion into the bilayer is often concomitant with secondary structure rearrangement in the peptide [19,20,21].

There are several physical models that were proposed for the membrane disruptive activity of AMPs. The barrel stave model involves the insertion of the peptide into the membrane in forming a purely proteinaceous pore [22], the toroidal pore model causes bending of the lipid membrane leading to the formation protein and lipid headgroup lined pore [23], and the carpet model which causes destabilization of the cell membrane through a mass action effect similar micellization type mechanism [24]. 

Many antimicrobial peptides are naturally occurring components of the innate immune system of higher organisms, while other naturally occurring peptides can also exhibit antimicrobial activity. Naturally occurring venoms and toxins often contain a cocktail of biological active molecules and are a rich source for the identification of naturally occurring peptides. Many of these peptides function naturally to damage the cell membrane as part of the mechanism of action of the venom. Well-characterized examples include, the honeybee venom peptides melittin [25], wasp peptides MP1 and mastoparan, and the rattlesnake toxin crotamine [26].

Antimicrobial peptides isolated from different species of fish have become a focus of research because fish are abundant in peptides as a defense mechanism after hatching [27,28]. Pleurocidins are a class of antimicrobial peptide which were isolated from a variety of Atlantic flounder species, including the winter flounder Pleuronectes americanus [27,29,30]. In this study, different analogs of the Pleurocidin like peptide AP3, from *P. americanus*, were synthesized to further investigate the sequence-structure and activity relationship in these peptides. Different analogs of AP3 were synthesized in which length, charge identity, and Trp position were varied.

This study focused on modified AP3 constructs in which the Trp position was changed to facilitate the creation of truncated sequences that still contain Trp as a fluorescent reporter for binding and depth studies as described previously [31,32]. Another full-length variant was analyzed, in which the single Arg was replaced with Lys to create uniform charge identity in the peptide. The peptides were characterized using fluorescence spectroscopy to analyze bilayer interactions, circular dichroism to determine secondary structure, and membrane permeabilization assays.

## 2. Results

### 2.1. Peptide Design and Characterization

**Figure 1:**
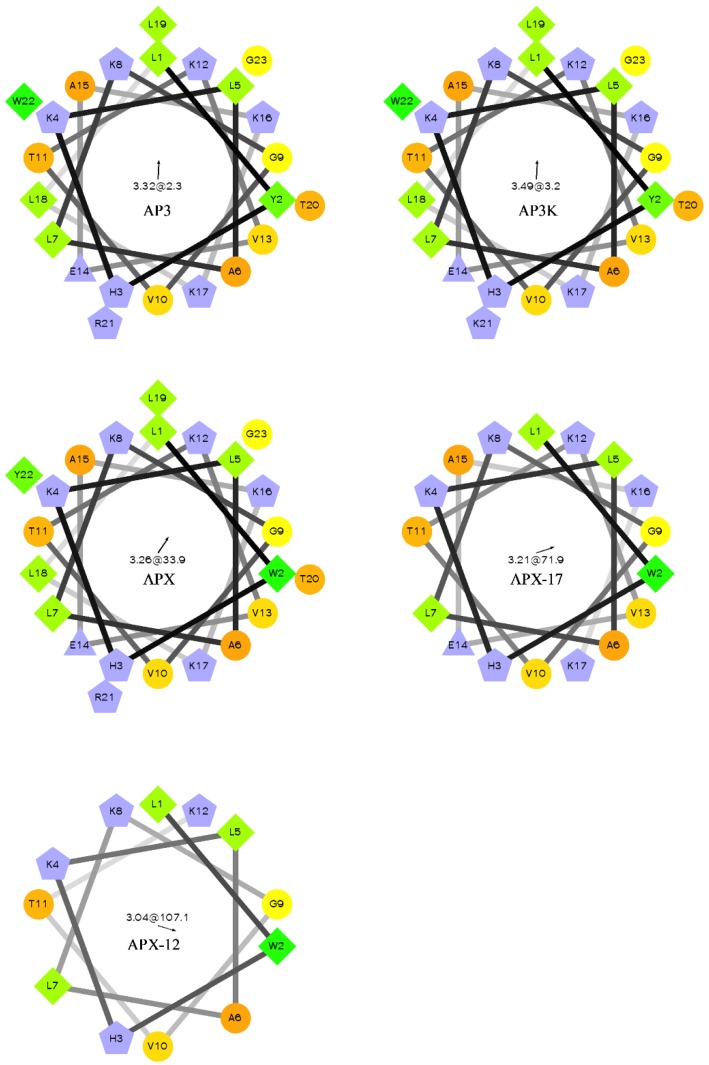
Helical wheel of AP3 and derivative sequences. Orange circles represent hydrophilic residues, green diamonds represent hydrophobic residues, blue triangles represent anionic, and blue pentagons represent cationic residues. The arrow and number at the center of the helical wheel is the direction and magnitude of the hydrophobic moment. Projections were made using http://rzlab.ucr.edu/scripts/wheel/wheel.cgi.

All peptides were based on the original Ap3 sequence (Table 1, Figure 1). In all cases, the sequences were modified or truncated, but retained a Trp residue to allow for fluorescence assays described below. APX and the truncated versions included a change in the position of the Trp residue to facilitate synthesis. Peptides were purified via reversed phase HPLC and identity was confirmed by ESI-MS (Table 1). Peptide stock concentrations were determined using absorbance spectroscopy.

### 2.2. Minimum Inhibitory Concentration (MIC)

The antibacterial activities of each peptide were determined against various Gram-negative and Gram-positive bacteria using minimum inhibitory concentration (MIC) assays. The MIC is defined as the lowest concentration of peptide that prevents growth of bacteria using the optical density at 600 nm under standard growth conditions [33]. The parent peptide AP3 shows equal or greater antimicrobial activity against all organisms tested when compared to the other full length and truncated analogs (Table 2), with the exception of a two-fold increase activity of AP3K against *S. aureus*. AP3 had the lowest MIC (highest activity) against *P. aeruginosa*, exhibiting an MIC of approximately 0.3 μM. The AP3K variety exhibited a similar profile to the parent AP3 with no more than a two-fold difference against any strain tested. Interestingly, the AP3 sequence was not as effective against a clinically isolated strain of *E. coli* [34]. APX variant showed very weak antimicrobial activity, only exhibiting killing at 16 μM for *S. aureus*. None of the truncated analogs exhibited antimicrobial activity against any of the strains tested at any of the concentrations indicated. 

### 2.3. Lipid Binding

**Figure 2:**
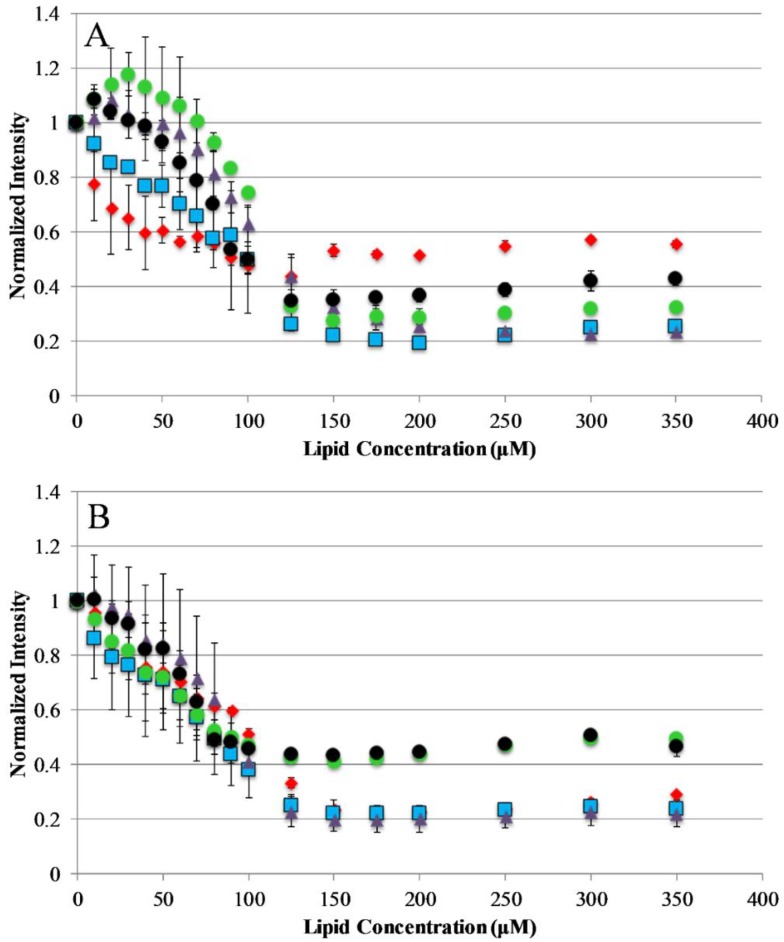
Peptide binding to lipid vesicles: Peptide interaction with lipid vesicles was monitored using Trp emission spectra. The normalized Trp emission intensity is shown for each AP peptide titrated with either 75/25% PCPG (**A**) or 100% PC (**B**) lipid vesicles. For both graphs, AP3 is shown as red diamonds, AP3K as blue squares, APX as purple triangles, APX-17 as black circles, and APX-12 as green circles. Peptide concentration was 2 μM in all cases. Data shown are corrected for background fluorescence and represent averages of at least three replicate samples. Error bars represent the standard deviation of these data.

The current understanding of antimicrobial peptide mechanism of action involves the binding of peptides to the bacterial membrane as the first step in the bactericidal activity. Binding experiments were designed to monitor the interactions of the AP peptides with model lipid bilayers. This was approached using tryptophan emission fluorescence spectroscopy due to the environmental sensitivity of tryptophan fluorescence. This sensitivity is often exploited to discriminate between the polar milieu and the more nonpolar lipid bilayer environments. Model vesicles were composed of either 100% PC or 75/25% PC/PG lipids to investigate the effect of electrostatics on the binding of peptides to bilayers. The electrostatic binding mechanism is widely accepted as a major component of the selectivity of antimicrobial peptides (bacterial membranes contain high mole fractions of anionic lipids imparting a strongly negative net charge to the cell surface) [35,36]. Interestingly, our results show that only the original parent sequence AP3 exhibited any significant shift in emission barycenter when interacting with lipid vesicles as shown in Supplemental Figures S1 and S2, Supplementary Materials. However, all peptides in this study exhibited significant decreases in emission intensity upon introduction of lipid vesicles, which is likely a result of binding interaction (Figure 2). Interestingly, none of the peptides in this study showed any significant preference between zwitterionic PC vesicles and vesicles composed of 75%/25% PC/PG. This is notable for the peptides which are truncated, such as APX-12, which have a reduced net charge. The extent of fluorescence intensity quenching on binding may also be impacted by the final conformation in the bound state. 

### 2.4. Fluorescence Quenching

**Figure 3:**
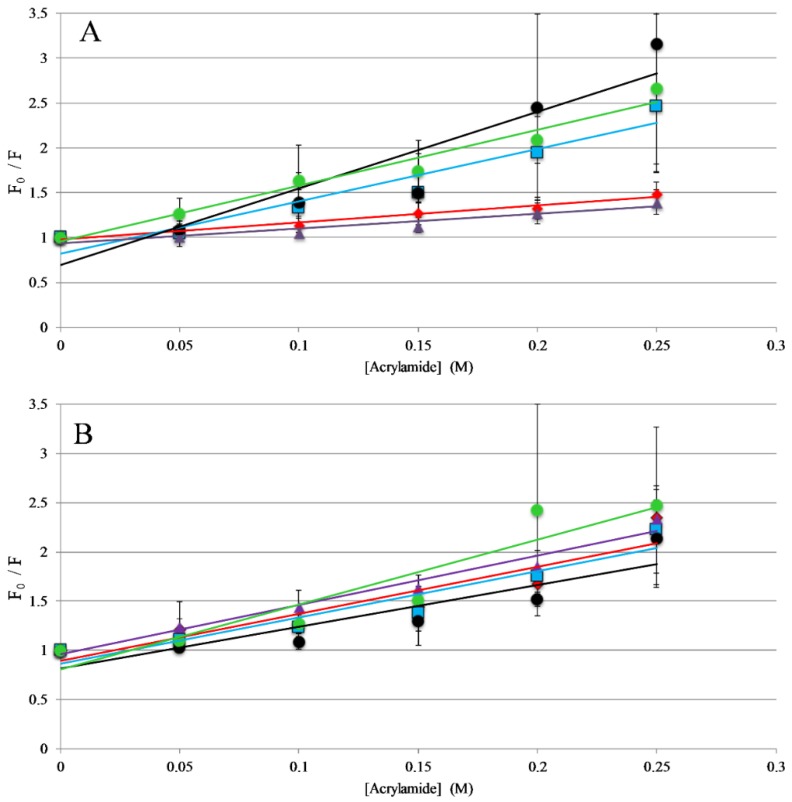
Trp Emission Quenching by Acrylamide: Fluorescence quenching of AP3 and derivatives by acrylamide (**A**) in the presence of 250 μM 75:25 PC: PG vesicles or (**B**) in the absence of lipid vesicles (in buffer solution). For both graphs, AP3 is shown as red diamonds, AP3K as blue squares, APX as purple triangles, APX-17 as black circles, and APX-12 as green circles. Lines represent linear fits of the data. Data shown are corrected for background fluorescence, inner filter effects, and are averages of at least three replicate samples. Error bars represent the standard deviation of these data.

The titrations described above indicate that all the peptides in this study interact with lipid membranes, while the topography and structural orientation of peptides associated with the bilayer may be different among the different peptides. Changes in peptide orientation, with respect to the bilayer, can impact fluorescence properties and can be related to the mechanism of action. Fluorescence quenching experiments were performed to gain insight on the orientation in the bilayer. Tryptophan fluorescence quenching was performed using the collisional quencher acrylamide, which can strongly quench tryptophan exposed to the aqueous environments. However, previous results show acrylamide causes very minimal quenching of tryptophan residues buried in the nonpolar core of the bilayer [31,37,38]. Quenching of the peptides was compared in the presence and absence of lipid vesicles. For the full-length peptides, AP3 and APX were more strongly quenched in the absence of lipid compared to the presence of lipid, indicating a greater degree of occlusion of the tryptophan when bound to the lipid bilayer (Figure 3, Table S1, Supplementary Materials). Interestingly, the AP3K peptide showed very little difference in quenching profile with and without lipid indicating that the AP3K peptide may adopt a different 3D topography when bound to the bilayer compared to AP3 and APX (Figure 3). Truncated analogs of APX show similar results to AP3K, in that there was no significant difference in quenching between the bound and unbound samples. This is indicative that, when bound, the AP3K and the truncated analogs adopt a structure, which orients the tryptophan more shallowly in the bilayer increasing the exposure to acrylamide in the aqueous environment.

### 2.5. Circular Dichroism (CD) Spectroscopy

**Figure 4:**
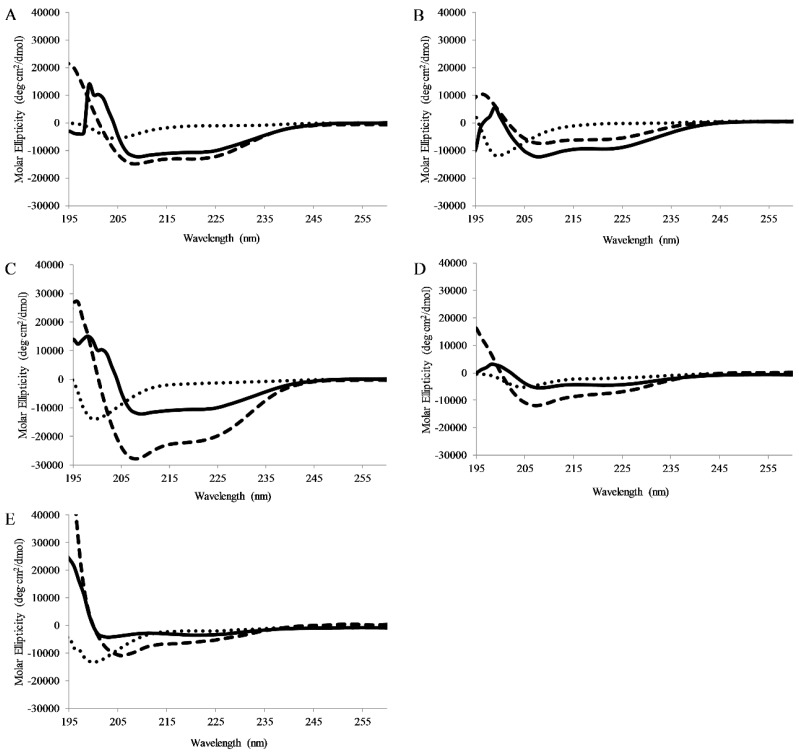
Circular Dichroism Spectroscopy: Circular dichroism spectra of 5 μM (**A**) AP3, (**B**) AP3K, (**C**) APX, (**D**) APX-17, or (**E**) APX-12 Spectra were recorded in the presence of pH7 buffer, 1:1 TFE:pH7 buffer, or 250 μM 75:25 PC:PG SUVs. For all graphs, peptide in buffer is shown as dots, peptide in buffer:TFE as dashes, and peptide with Lipid vesicles as solid lines. All spectra are averages of 64 scans and were background subtracted.

CD spectra were collected for peptides dissolved in pH 7 buffer, pH 7 buffer with lipid vesicles (75/25% PC/PG), or pH 7 buffer and TFE (50/50 buffer/TFE). TFE is known to promote helical structure and is used as a control. In buffer all peptides exhibited CD signatures consistent with random coil structure (minima ~198nm). When bound to lipid vesicles, all of the full-length peptides displayed CD spectral consistent with the formation of α-helical secondary structure (minima 208 and 222 nm) (Figure 4). Interestingly, APX-17 showed less helical character than the shorter APX-12 which was clearly adopting helical structure. As anticipated, all peptides were shown to adopt a helical conformation when in the presence of TFE.

### 2.6. Lipid Vesicle Permeabilization

**Figure 5:**
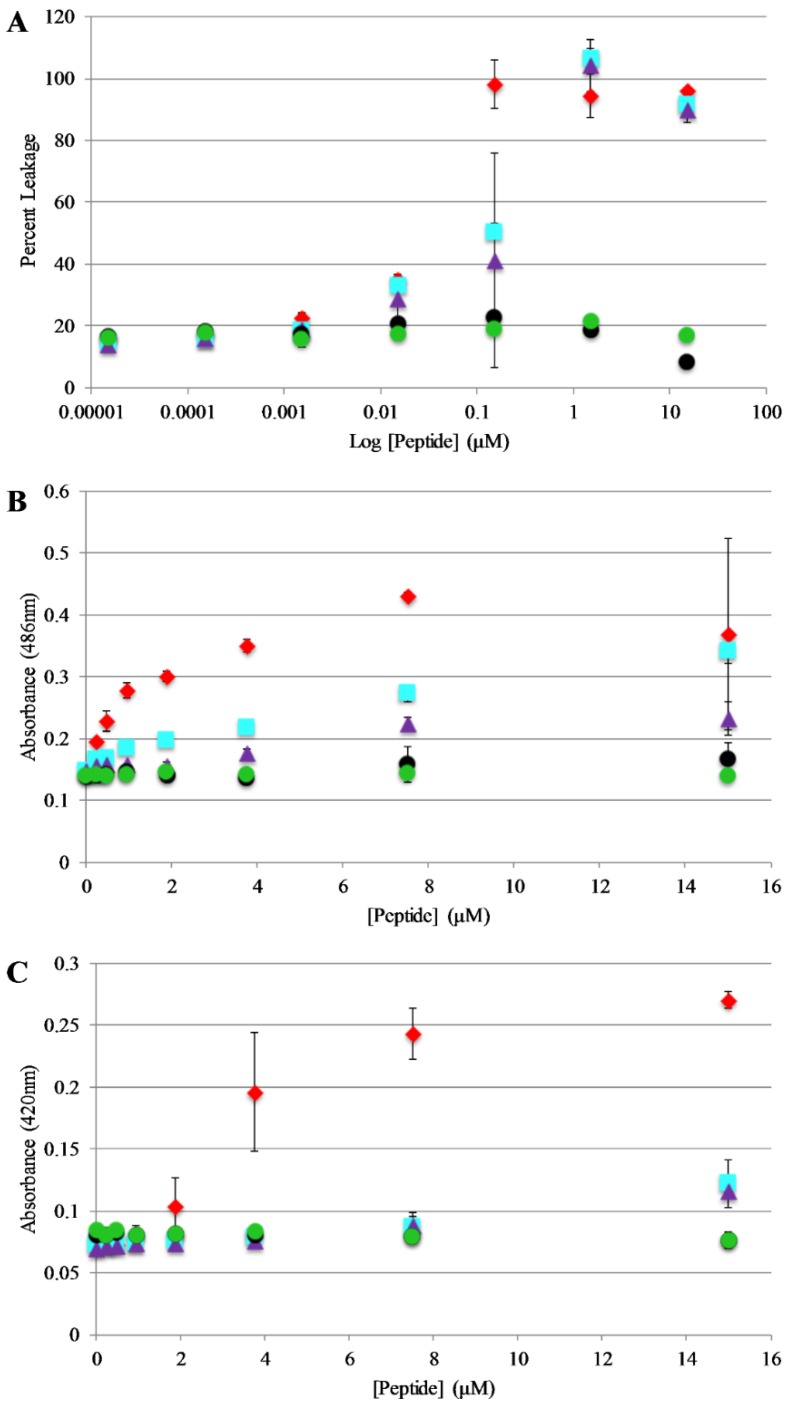
Vesicle and Bacterial permeabilization: (**A**) Leakage of calcein entrapped in 75:25 PC:PG lipid vesicles (200 μM) after exposure to peptides. Percent leakage was calculated by treating each sample with Triton X-100 and remeasuring fluorescence which was then set to 100% leakage. (**B**) Outer membrane permeability of E. coli after treatment with peptides. Samples contained 50 μg/mL nitrocefin, and 80 µL of resuspended *E. coli* cell suspension, and 10 μL peptide from a stock solution for the appropriate final concentration. Absorbance values shown represent data collected after 30 min of exposure to peptide. (**C**) Inner membrane permeability of *E. coli* after treatment with peptides. Samples contained 56.25 μL Z-buffer (100 mM Na_2_HPO_4_, 10 mM KCl, 1 mM MgSO_4_, 40 mM β-mercaptoethanol, pH 7.1), 12 μL ortho-Nitrophenyl-β-galactoside (ONPG) (4 mg/mL), 18.75 μL *E. coli*, (5.0 × 10^5^ cfu/mL) and 10 μL of peptide from the appropriate final concentrations. Absorbance values shown represents data collected after 30 min of exposure of peptide. For all graphs AP3 is shown as red diamonds, AP3K as blue squares, APX as purple triangles, APX-17 as black circles, and APX-12 as green circles. Data are averages of at least three replicate samples. Error bars represent the standard deviation of these data.

Bilayer disruption and/or pore formation is commonly proposed to be a component of the mechanism of action of antimicrobial peptides. Calcein leakage assays were performed to determine if AP3 and analogs are capable of causing bilayer disruption, allowing molecules to cross the membrane [39,40]. In this assay, the fluorophore calcein is entrapped within lipid vesicles where fluorescence emission is strongly quenched through a self-quenching mechanism. If peptides induce bilayer disruption or create sufficiently large pores in the bilayer, the entrapped calcein can leak out of the vesicle lumen thus relieving the self-quenching and resulting in an increased in calcein fluorescence emission intensity. Leakage assays were performed using 75%/25% PC/PG vesicles and leakage was monitored over the course of 30 min. However, the samples reached a steady state after no more than 5 min in all cases. Full release was determined by adding an aliquot of the detergent Triton X-100 to each well after the 30 min time course. The full-length peptides exhibit similar dose dependent vesicle permeabilization activity with near complete permeabilization achieved between 0.1–1 μM peptide (Figure 5A). Notably, the truncated peptides induced minimal if any leakage even at the highest concentrations tested.

### 2.7. Bacterial Membrane Permeabilization

Based on the results of the calcein leakage assays, the permeabilization of live, intact *E. coli* was investigated using enzyme-based leakage assays. Briefly, these assays rely on limited diffusion of a chromogenic substrate across a bacterial membrane(s). If the membrane(s) are disrupted by an antimicrobial peptide (or other agent) the substrate can more easily transit across the membrane(s) where it can be broken down by a bacterially expressed enzyme. The assay for outer membrane permeabilization relies on the enzyme β-lactamase, located in the *E. coli* periplasmic space, which can cleave the β-lactam containing molecule nitrocefin. The nitrocefin break down results in a chromogenic product [32,41]. Bacteria were exposed to varying concentrations of the AP peptides and nitrocefin cleavage was monitored over a 90 min time course. Figure 5B represents the data collected after 30 min of the assay (this was after samples had reached steady state. Figure S3, Supplementary Materials, full-time course). The parent peptide AP3 exhibited the highest degree of bacterial membrane disruption followed closely by the other full-length peptides. Consistent with the calcein leakage data, the truncated peptides showed little to no disruption at any of the concentrations tested. 

The assay for inner membrane permeabilization relies on the enzyme β-galactosidase, located in the E. coli inner membrane, which can cleave the molecule ONPG a chromogenic mimic of the natural substrate lactose. Similarly, the ONPG cleavage results in a chromogenic product [32,41]. Bacteria were exposed to varying concentrations of the AP peptides and ONPG break down was monitored over a 90 min time course. Figure 5C represents the data collected after 30 min of the assay (this was after samples had reached steady state, Figure S4, Supplementary Materials full-time course). Consistent with the results of the outer membrane permeabilization experiments, the parent peptide AP3 exhibited the highest degree of bacterial membrane disruption followed closely by the other full-length peptides and little-to-no disruption induced by the truncated peptides.

### 2.8. Mammalian Cell Viability

**Figure 6:**
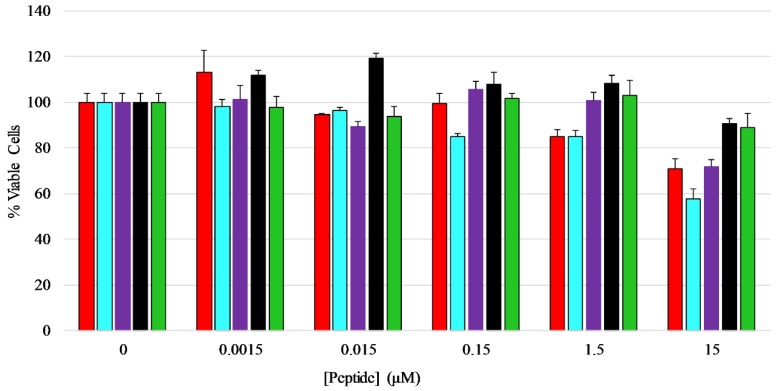
Mammalian cell viability assay—Effects of AP3 peptides on HEK-293 cell viability assayed by MTT metabolic breakdown. A standard density of HEK-293 cells was incubated with serially diluted peptides for 24 h at 37 °C before the assay was performed. Percent viability was normalized to MTT breakdown observed in untreated controls. Color coding of the graph is AP3 (red), AP3K (blue), APX (purple), APX-17 (black), and APX-12 (green). All data represent the average of at least three replicates and error bars represent the standard deviation.

An MTT assay was performed to determine if the peptides exhibited any toxicity toward mammalian cells, in this case HEK-293 cells. The cells were treated with five different concentrations of each peptide ranging from 0.0015 µM to 15 µM for a total of 24 h before viability measurements were performed. The results show that the peptides did not have any major inhibitory effect on the growth or viability of HEK-293 cells over the majority of concentrations tested, however the full length peptides induced 20–40% loss in viability at the highest concentration tested (Figure 6) [42,43,44], with AP3K showing the highest extent of cytotoxicity (~40% dead cells) at 15 µM peptide, while both AP3 and AP3K exhibited some cytotoxic activity (~18% dead cells) at 1.5 µM peptide. 

## 3. Discussion

Antimicrobial resistance has become a global problem and the need for a solution minimizing the number of resistant bacteria is crucial [45]. Finding alternatives to the use of antibiotics is a strategy that has been used to try and minimize the problem of resistance. Antimicrobial peptides are one class of molecules that were investigated due to its low propensity for resistance along with its ability to kill or inhibit bacterial growth [2,8]. We attempted to use this strategy to further explore a relatively unknown antimicrobial peptide along with various analogs.

The data indicated that all the peptides in this study interacted with lipid bilayers with similar apparent affinities. This is interesting because, unlike many cationic AMPs, the inclusion of anionic lipids (POPG, in our study) did not have a significant effect on lipid binding. Numerous reports indicate the increase in anionic lipid concentration increases the affinity for peptides for lipid bilayers [46,47,48,49]. The binding enhancement cause by inclusion of anionic lipid in a bilayer is driven electrostatic interactions between cationic AMP and the anionic lipid surface, which mimics the bacterial cell surface [6]. Additionally, penetratin and its truncates showed preferential binding to POPC/POPG membranes over DMPC/DMPG bilayers, indicating a bilayer thickness effect [50]. The ability for peptides to span the bilayer is thought to be a component of, or at least related to, the ability to disrupt lipid bilayer integrity and, thus, a link to hydrophobic matching is not unexpected [8,47,51]. 

Circular dichroism results indicate a clear formation of α-helical secondary structure for all AP3 peptides examined when they were bound to model lipid bilayers. This is consistent with numerous reports in the literature, indicating a random coil to helical conformation change for cationic AMPs upon binding to lipid bilayers [32,52]. Interestingly, the truncated peptides also adopted an α-helical conformation when bound to a lipid bilayer, although to a lesser extent [53]. This indicates that the loss of hydrophobic and cationic groups does not completely interfere with the ability of these peptides to adopt α-helical secondary structure [53].

For most antimicrobial peptides, there is a clear facially amphiphilic structure when the peptide adopts an α-helical conformation. Generally, cationic residues segregate to one face of the helix where hydrophobic moieties segregate to the opposite face [54,55,56]. However, there is very little description in the literature of peptides which exhibits antimicrobial activity but do not adopt facially amphiphilic structures. Many reports focus on the facially amphiphilic structure as a necessary component of antimicrobial activity in peptides and polymers, often using non-facially amphiphilic controls which exhibit decreased efficacy. As shown in Figure 1, the AP3 and derivatives peptides do not show any facial amphiphilicity. This could be tied to the apparent insensitivity to anionic lipid composition, which may in part result from an active conformation that cannot efficiently bury hydrophobics, such as the reporter Trp, in the bilayer core. As the peptides are truncated, they lose both hydrophobic and cationic residues, but this lose does not significantly affect the overall distribution of groups around the peptide helical axis. As such, it appears the hydrophobic burial interactions drive the binding of these peptides to the bilayer.

The binding to lipid membranes and helix formation in the AP3 peptides indicates that they are behaving somewhat similar to traditional amphiphilic AMPs. However, the lack of facial amphiphilicity presents an interesting question regarding how these peptides orient on/in the lipid bilayer. The lack of facial amphiphilicity makes the partition of traditional hydrophobic residue into the lipid bilayer difficult without also resulting in some burial of some hydrophilic groups. Acrylamide, an aqueous quencher of tryptophan fluorescence, was used to assay the orientation and topology of the AP3 peptides when bound to lipid bilayers. Strikingly, the full-length peptides exhibited significantly different quenching patterns compared to the truncated peptides. This indicates that while all the peptides sequences studied bind to lipid bilayers, the final conformation at the bilayer surface is clearly different. Notably, the APX peptide series have the Trp at position 2 while the AP3 and AP3K have the Trp reporter at position 22, meaning that in either a transmembrane or surface orientation, the Trp residue should be somewhat exposed to the aqueous milieu. The observed differences could be attributed to altering the charge/hydrophobic balance or as a result of hydrophobic mismatch between the shorter peptides and the bilayer. 

Bacterial membrane permeabilization and vesicle leakage assays yielded consistent results in that full-length peptides caused a greater extent of leakage across the bilayer(s) compared to the truncated peptides. These results indicate that altering the length and charge of an antimicrobial peptide can significantly lower the antimicrobial effectiveness, and the mechanism of action may be potentially affected, as reported elsewhere [57,58,59]. Depending on the mechanism, the shortened length of the truncated peptides may impact the ability to disrupt bilayers due to effects arising from hydrophobic mismatch [59,60]. 

The MIC assay performed indicated the truncated peptides are less effective as antimicrobial agent compared to the full-length sequence, which is consistent with recent reports in literature [61,62,63]. However, antimicrobial activity for AMPs is not exclusively linked to peptide length, as many naturally occurring AMPs are as short as the shortest sequences tested here [64,65]. Similarly, reports in the literature found that decreasing the overall net charge of cationic AMPs causes the efficacy to also be frequently reduced [57]. The lack of antimicrobial activity for the truncated peptides in this study is not surprising considering the inability to permeabilize bacterial membranes. 

The AP3 derived peptides also showed relatively low cytotoxic activity except at the highest concentrations tested. This is consistent with the membrane permeabilization activity assays. However, the high concentrations required for antibacterial activity indicate there may be limited selectivity in these peptides. Many studies linked the cytotoxic effects of AMPs and AMP mimetics to hydrophobic interactions, in which increased hydrophobic content is proportional to increased cytotoxicity [16,17,18,66,67]. Notably, the peptides with the best antimicrobial activity, AP3 and AP3K, were also the most cytotoxic.

The AP3 and APX peptide comparison is of interest as the only difference between these two peptides is the location of the Trp, yet there was a four-fold difference in *S. aureus* MIC values and at least a two-fold difference in *E. coli* MIC values. They seem to bind and quench similarly but have different permeabilization. This is surprising considering the small differences in the quenching and binding. The behavior in the leakage assay seems to mimic the behavior in the MIC assay more closely than the binding or the quenching assays. This reinforces the value of extending biophysical studies, as well as natural membrane studies, to better recapitulate the environment the peptides would encounter.

## 4. Materials and Methods

### 4.1. Peptide Preparation

The peptides APX, APX-17, and APX-12 were prepared by FMOC solid phase synthesis. Synthesis was performed on rink-amide resin using Fmoc-protected amino acids, HATU (1-[Bis(dimethylamino)methylene]-1H-1,2,3-triazolo[4,5-b]pyridinium 3-oxid hexafluorophosphate), and DIEA (N,N-Diisopropylethylamine) at a 1:1:2 ratio for couplings. Deprotections were performed using a solution of 1:4 v:v: piperidine in DMF (N,N-Dimethylformamide). Cleavage was performed by mixing the resin with a cocktail of TFA (trifluoroacetic acid):TIS (triisopropylsilane):H2O:ethanedithiol (92.5:2.5:2.5:2.5) for ~2 h followed by filtration and peptide precipitation in cold diethyl ether. Remaining peptides were purchased from GenScript (Piscataway, NJ, USA). All peptides were purified by reverse phase high-performance liquid chromatography using a linear gradient of solvent A (H20 with 0.1% TFA) and solvent B (acetonitrile with 0.1% TFA). Peptides were separated on an Agilent (5 μm 9.4 × 250 mm) C4 column. Fractions were monitored using UV absorbance at 220 and 280 nm and peptide identity was confirmed using ESI-MS (Agilent 1100 Series LC/MSD Trap, Santa Clara, CA, USA). Purified peptides were lyophilized, reconstituted in an ethanol water mixture and stored at 4 °C. Peptide stock concentration was determined spectrophotometrically, as described previously [31,32].

### 4.2. Bacterial Culturing

Bacteria were streaked from frozen stocks onto Difco LB-miller agar (BD, Franklin Lakes, NJ, USA) plates (*E. coli* MG1655 ATCC 47076, *E. coli* D31 [34], *S. aureus* ATCC25923, *P. aeruginosa* ATCC10145, *K. pneumoniae* 700603). Streaked plates were grown overnight at 37 °C and subsequently stored at 4 °C.

To prepare overnight cultures, a single colony of each bacterial strain was added to LB broth (Difco) in sterile culture tubes. Tubes were placed in the shaking incubator at 37 °C overnight to allow for sufficient bacterial growth. After incubation, dilutions of the overnight culture were made in LB (1:100) or Muller-Hinton broth (Criterion) (1:250) for MIC assay protocols (Hardy Diagnostics, Santa Maria, CA, USA).

### 4.3. Minimum Inhibitory Assay (MIC)

Minimal inhibitory concentrations (MIC) were determined for each peptide The MIC assay was set up by adding 10 µL of the peptide stock solutions to wells of a sterile 96-well plate to which 90 mL of diluted bacterial culture was added. The final bacterial load was ~5.0 × 10^5^ cfu/mL as calculated from the Optical Density^@^ 600 nm (OD_600_) measurements of a log-phase culture. The assay plate was incubated at 37 °C for 18 h. Bacterial growth was assayed by absorbance at OD_600_ using Multiskan microplate reader (Thermo Fisher, Waltham, MA, USA). The MIC was defined as the lowest peptide concentration to completely inhibit growth as judged by the absorbance at 600 nm compared to controls. All MIC values are averages of at least triplicate samples.

### 4.4. Lipid Preparation

Stocks of PC (1-palmitoyl-2-oleoyl-sn-glycero-3-phosphocholine, POPC) and PG (1-palmitoyl-2-oleoyl-sn-glycero-3-phospho-(1’-rac-glycerol), POPG) (Avanti Polar Lipids, Alabaster, AL, USA) were dissolved in chloroform and stored at −20 °C. Vesicles were prepared by mixing appropriate aliquots of lipids in a glass test tube which were dried first with nitrogen gas and then under a vacuum for 1 h. Small unilamellar vesicles (SUVs) were formed by resuspending the lipid film in PBS buffer (50 mM sodium phosphate, 100 mM NaCl pH 7.1) before sonicating in a high-power bath sonicator for 18 min (Avanti Polar Lipids).

### 4.5. Binding Experiments

Peptide binding to lipid vesicles was determined using fluorescence spectroscopy by titrating in aliquots of lipid vesicles into a sample that contained buffer and peptide. Lipid vesicles were prepared as described above, with either 100% PC or 75:25 PC: PG (PC/PG) lipid composition and final peptide concentration of 2 μM. Trp Emission spectra were recorded from 300 to 400 nm with an excitation of 280 nm using a Spectramax-2 spectrofluorometer (JY-Horiba, Edison, NJ, USA). All spectra were recorded at 25 °C.

### 4.6. Acrylamide Quenching

Samples for acrylamide quenching analysis composed of 250 μM total lipid and 2 μM peptide. Samples were excited at 295 nm with emission measured over the range of 315–415 nm. Fluorescence measurements were taken prior to addition of quencher as the F_0_ value. Aliquots from a 4 M stock of acrylamide in water were added to the samples, mixed, and then remeasured. All spectra were recorded at 25 °C. Fluorescence from background samples containing only lipid vesicles, buffer and quenchers were subtracted. Emission intensity was also corrected for dilution by addition of the quencher and for inner filter effects by the quencher as described previously [31].

### 4.7. Circular Dichroism

Samples were prepared in a buffer containing 1:10 diluted PBS (5 mM phosphate, 10 mM NaCl, pH 7.04) with peptide concentration of 5 μM unless specified otherwise. A Jasco J-715 CD spectrometer was used to record circular dichroism (CD) spectra using a quartz cuvette with a 1 cm path length. All spectra were recorded at 25 °C and averaged over 64 scans.

### 4.8. Calcein Leakage Assay

Lipid films were prepared as described as above for a final concentration of 20 mM (PC /PG). Lipid films were rehydrated with 46 mM calcein in 33 mM HEPES, pH 7. After being fully dissolved, the sample was subjected to 11 freeze-thaw cycles by transferring between liquid nitrogen and a 37 °C water bath. Vesicles were then extruded by passing the solution through 0.2 μM polycarbonate filters for 21 passes to create large unilamellar vesicles (LUVs). Extruded LUVs were separated from free calcein using size exclusion column chromatography (Sephadex G-100). Lipid concentrations and dilution factors were determined spectroscopically using a fluorescent reporter lipid. Assays were performed in a 96-well plate, which was set up such that each well contained lipid vesicles to a final concentration of 150 µM, 15 μL peptide diluted from a stock for appropriate final concentration in the wells, and HEPES buffer to a final volume of 150 μL. Complete calcein release was achieved by adding 5 μL of 170 mM Triton X-100 to each well at the end of the experiment. All data were normalized to the 100% release value according to the intensity after the Triton X-100 treatment. All experiments were performed at least in triplicate.

### 4.9. Bacterial Outer Membrane Permeabilization Assay

Bacterial culturing was performed as described above with the exception that *E. coli* (*E. coli* D31) was streaked onto LB-agar plates supplemented with 100 μg/mL ampicillin. The overnight cultures and dilutions were grown in the presence of ampicillin at a final concentration of 100 μg/mL. After subcultures were grown to OD_600_ ~0.2–0.3, cells were then centrifuged at 2500 rpm for 15 min and pellet was resuspended in PBS (100mM phosphate, 200mM NaCl, pH 7.0). The permeabilization assays were carried out using a 96-well clear flat bottom plate with wells containing 10 μL of 500 μg/mL nitrocefin, 80 μL of resuspended *E. coli* cell suspension, and 10 μL of peptide at various concentrations. A 0.25 mM solution of polymyxin B sulfate was used as a positive control. Immediately after addition of peptide the nitrocefin cleavage was monitored by absorbance at 486 nm in 5 min intervals for 1.5 h. Experiments were performed at least in triplicate.

### 4.10. Bacterial Inner Membrane Permeabilization Assay

Bacterial culturing was performed as mentioned above with the exception that *E. coli* (D31) was used and 50 μL of 100 mM IPTG was added to the cultures after dilution. The permeabilization assays were carried out using a 96-well clear flat bottom plate with wells containing 56.25 μL Z-buffer (100 mM Na_2_HPO_4_, 10 mM KCl, 1 mM MgSO_4_, 40 mM β–mercaptoethanol, pH 7.1), 12 μL ONPG (4 mg/mL), 18.75 μL *E. coli*, (5.0 × 105 cfu/mL), and 10 μL of peptide or the cationic detergent CTAB as a control. After these additions the ONPG cleavage was monitored by absorbance at 420 nm in 5 min intervals for 1.5 h. All experiments were performed at least in triplicate.

### 4.11. Measurement of Cell Viability

Cellular toxicity of specific peptides was determined by MTT ((3-(4,5-Dimethylthiazol-2-yl)-2,5-Diphenyltetrazolium Bromide)) assay. Human embryonic kidney (HEK) 293 cells with a lenti-CRE luciferase reporter gene were seeded in 96-well plates at a density of 1.5 × 10^4^ cells per well and incubated with 5% CO_2_ at 37 °C for 24 h. Then, cells were treated with appropriate concentrations of peptides (0.0015–15 µM) and control for 24 h. After treatment for 24 h, 10 µL of MTT solution (5 mg MTT/mL PBS) was added to all wells and incubated for 4 h. Then, DMSO was added as a solubilizing agent and the absorbance was recorded at 540 nm. Each peptide concentration was performed in triplicate.

## 5. Conclusions

From the combination of the results, the AP3 and derived peptides appear to bind to bilayers via hydrophobic interactions, but these hydrophobic interactions are not sufficient to cause significant cytotoxicity to mammalian cells or antibacterial activity in some cases. Additionally, binding to model lipid vesicles did not predict antimicrobial activity nearly as well as leakage assays. These results highlight the delicate balance in structure activity relationships among antimicrobial peptides and warrant more in-depth investigations.

## Figures and Tables

**Table 1:** Peptide Sequences and Properties.

Peptide	Sequence	Molecular Weight		Net Charge	pI
		Measured	Calculated		
AP3	GWRTLLKKAEVKTVGKLALKHYL	2652.8	2653.2	+5	10.89
AP3K	GWKTLLKKAEVKTVGKLALKHYL	2617.8	2625.2	+5	10.74
APX	GYRTLLKKAEVKTVGKLALKHWL	2653.2	2653.2	+5	10.89
APX-17	KKAEVKTVGKLALKHWL	1950.2	1949.3	+4	10.85
APX-12	KTVGKLALKHWL	1393.7	1394.7	+3	10.98

**Table 2:** MIC (μM).

Peptide	*S. aureus*	*E. coli*	*P. aeruginosa*	*K. pneumoniae*
AP3	4.15	8.3	0.25	4.15
AP3K	2.32	9.3	0.29	>9.30
APX	15	>15.0	15	>15.0
APX-12	>15.0	>15.0	>15.0	>15.0
APX-17	>8.00	>8.00	>8.00	>8.00

## Supplementary Materials

The following are available online at https://www.mdpi.com/2079-6382/8/1/20/s1, Figure S1: Trp emission spectra of peptides. Figure S2: Peptide binding to Lipid Vesicles by Spectral shifts. Figure S3: Time course of Outer-membrane permeabilization. Figure S4: Time course of Inner-membrane permeabilization. Table S1: Acrylamide Ksv.
