# Supplementary material for: Characterization and Antimicrobial Activity of Amphiphilic Peptide AP3 and Derivative Sequences
Source: Antibiotics (Basel). 2019 Mar 6;8(1):20. doi: 10.3390/antibiotics8010020 (PMC6466588; doi:10.3390/antibiotics8010020)
Supplement: Supplementary file 1 [file antibiotics-08-00020-s001.pdf]

antibiotics

MDPI

Article

Characterization and Antimicrobial Activity of Amphiphilic Peptide AP3 and Derivative Sequences

Christina L. Chrom [1], Lindsay M. Renn [1] and Gregory A. Caputo [1,2,*]

[1] Department of Chemistry and Biochemistry, Rowan University, Glassboro NJ 08028, USA; chromc95@rowan.edu (C.L.C.); rennl28@rowan.edu (L.M.R.)

[2] Department of Molecular and Cellular Biosciences, Rowan University, Glassboro NJ 08028, USA

* Correspondence: caputo@rowan.edu

Supplementary Materials

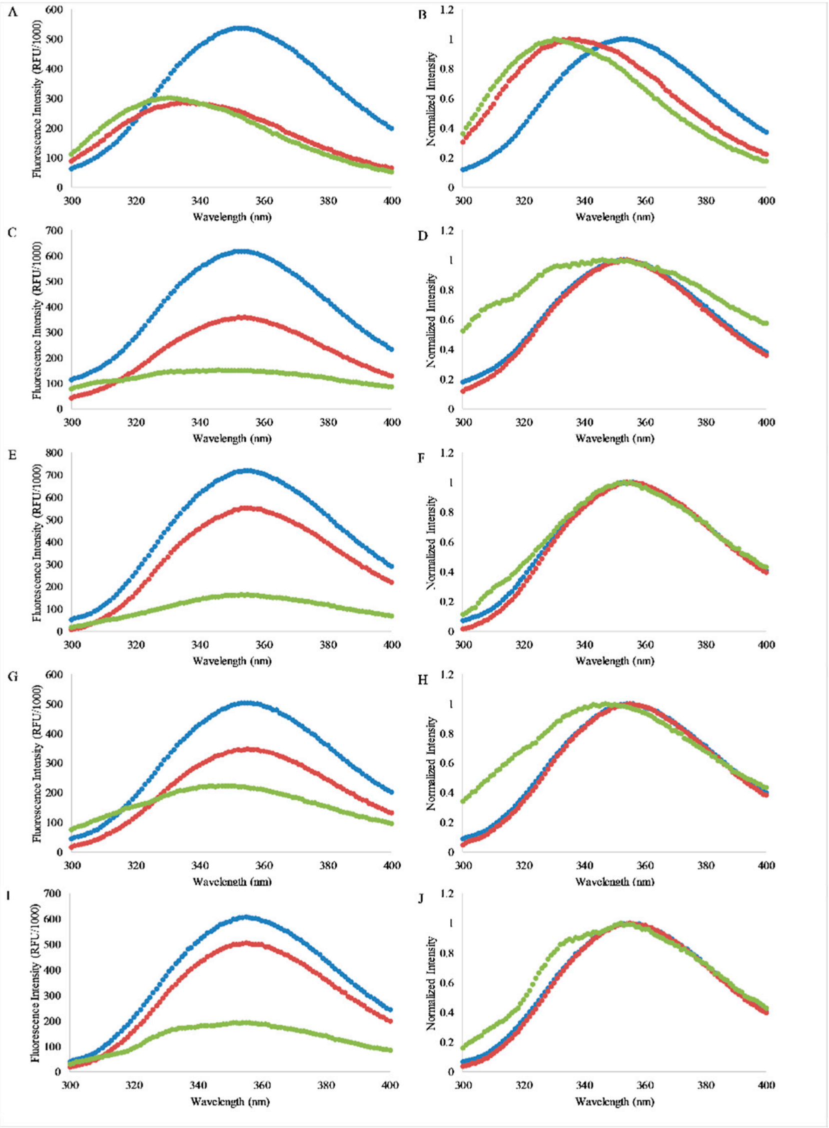

Figure S1. Trp emission spectra of peptides in buffer (blue), bound to 90 M PC:PG vesicles (red), or bound to 300 M PC:PG vesicles. The left column shows raw intensities while the right column shows normalized intensity for ease of comparison for spectral shifts. (**A**,**B**) AP3, (**C**,**D**) AP3K, (E,F) APX, (G,H) APX-17, (I,J) APX-12. Peptide concentration was 2 µM in all cases. Representative spectra are shown after correction for background fluorescence.

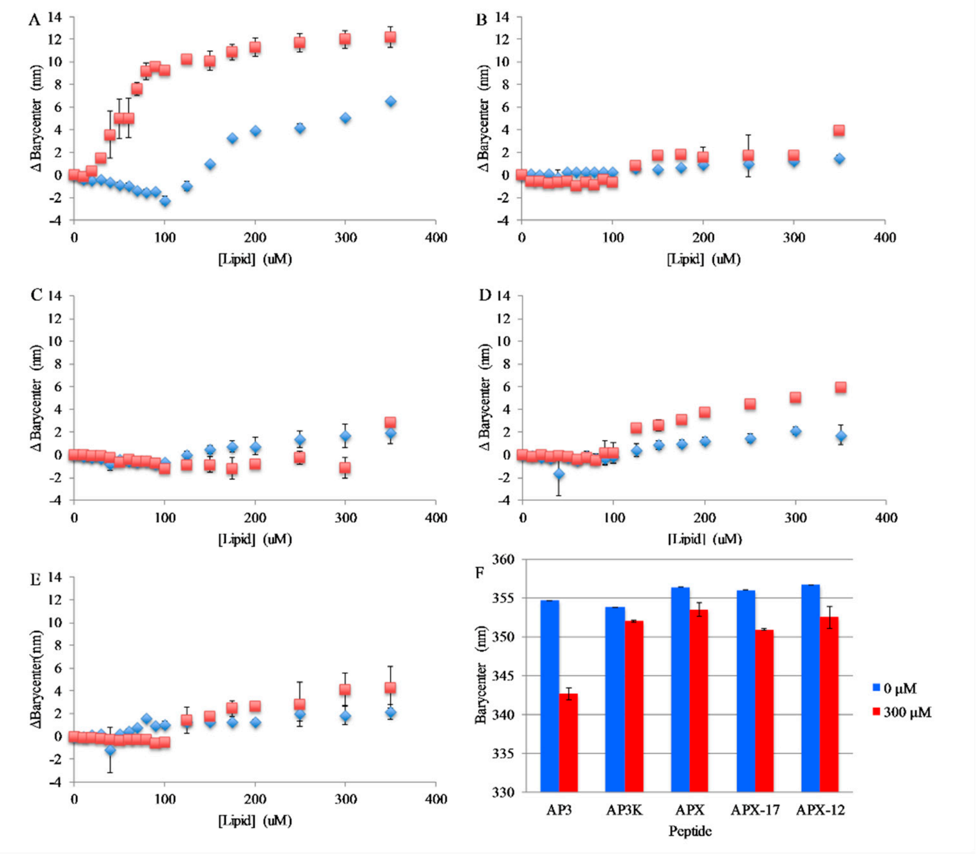

Figure S2. Peptide binding to Lipid Vesicles by Spectral shifts—Binding of AP peptides to lipid vesicles analyzed by change in emission spectrum barycenter as a function of lipid concentration. (**A**) AP3, (**B**) AP3K, (**C**) APX, (**D**) APX-17, (**E**) APX-12. Each peptide was titrated with either 75:25 PC:PG (red squares) or 100% PC (blue diamonds) vesicles. (F) Barycenter comparison of peptides in solution (blue) and bound to 300 μM PC:PG vesicles (red). Peptide concentration was 2 μM in all cases. Data shown are corrected for background fluorescence and represent averages of at least three replicate samples.

Table 1. Acrylamide Ksv (M^{-1}, cm^{-1}) [a].

Peptide	*Buffer*	*PC/PG*
AP3	4.78	1.92
AP3K	5.82	4.71
APX	4.99	1.61
APX-12	6.61	6.21
APX-17	4.22	8.61

a. Ksv values derived from the linear fits of acrylamide quenching shown in Figure 3.

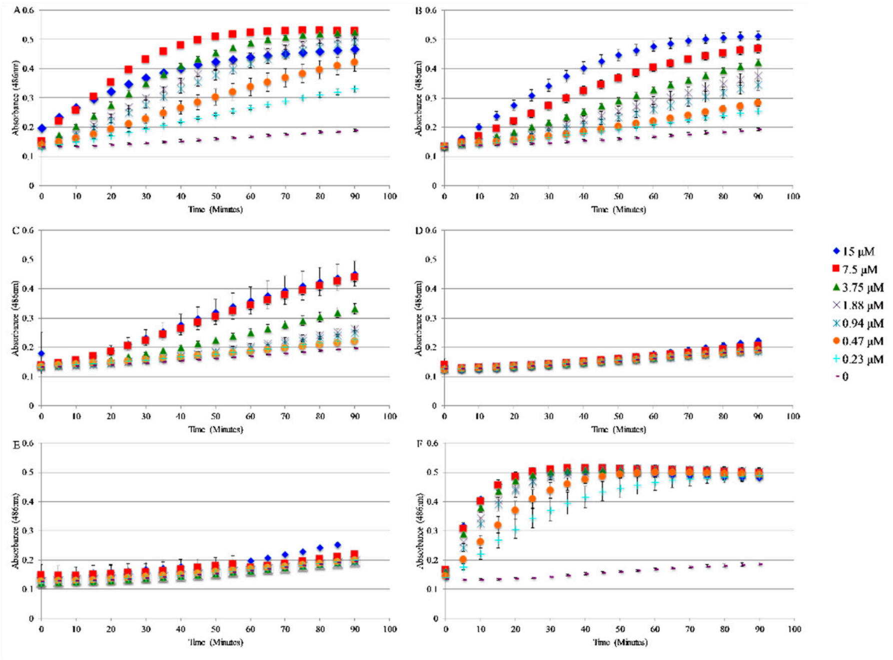

Figure S3. Time course of Outer-membrane permeabilization: Outer-membrane permeability of *E. coli* after treatment with peptides (A) AP3, (B) AP3K, (C) APX, (D) APX-17, (E) APX-12 or (F) the control Polymyxin B sulfate. For all graphs peptide concentration is shown in the legend. Samples contained 50µg/mL nitrocefin, and 80uL of resuspended *E. coli* cell suspension, and 10 µL peptide from a stock solution for the appropriate final concentration. Absorbance values shown represent data collected over 90 minutes of exposure to peptide. Data are averages of at least three replicate samples.

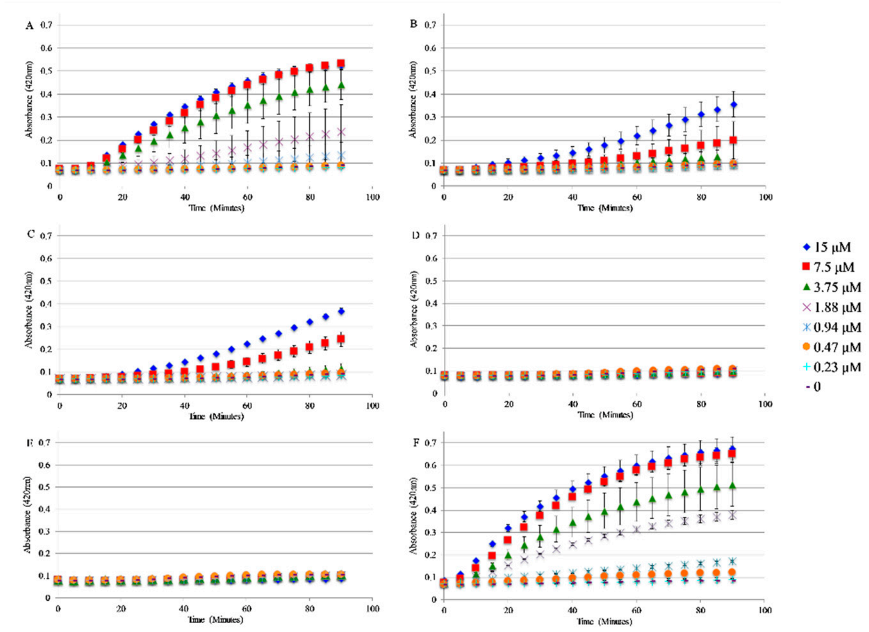

Figure S4. Time course of Iuter-membrane permeabilization: Inner-membrane permeability of *E. coli* after treatment with peptides (A) AP3, (B) AP3K, (C) APX, (D) APX-17, (E) APX-12 or (F) the control detergent CTAB. For all graphs peptide concentration is shown in the legend. Samples contained 56.25uL Z-buffer (100 mM Na_2HPO_4, 10 mM KCl, 1 mM $MgSO_4$, 40 mM β– mercaptoethanol, pH 7.1), 12µL ONPG (4mg/ml), 18.75uL E. coli, (5.0 x 10^5 cfu/ml) and 10µL of peptide from the appropriate final concentrations. Absorbance values shown represents data collected after 90 minutes of exposure of peptide. Data are averages of at least three replicate samples.
